# Assessing novelty, feasibility and value of creative ideas with an unsupervised approach using GPT‐4

**DOI:** 10.1111/bjop.12720

**Published:** 2024-07-22

**Authors:** Felix B. Kern, Chien‐Te Wu, Zenas C. Chao

**Affiliations:** ^1^ International Research Center for Neurointelligence (WPI‐IRCN) UTIAS, The University of Tokyo Tokyo Japan

**Keywords:** AUT, creativity, large language models

## Abstract

Creativity is defined by three key factors: novelty, feasibility and value. While many creativity tests focus primarily on novelty, they often neglect feasibility and value, thereby limiting their reflection of real‐world creativity. In this study, we employ GPT‐4, a large language model, to assess these three dimensions in a Japanese‐language Alternative Uses Test (AUT). Using a crowdsourced evaluation method, we acquire ground truth data for 30 question items and test various GPT prompt designs. Our findings show that asking for multiple responses in a single prompt, using an ‘explain first, rate later’ design, is both cost‐effective and accurate (*r* = .62, .59 and .33 for novelty, feasibility and value, respectively). Moreover, our method offers comparable accuracy to existing methods in assessing novelty, without the need for training data. We also evaluate additional models such as GPT‐4 Turbo, GPT‐4 Omni and Claude 3.5 Sonnet. Comparable performance across these models demonstrates the universal applicability of our prompt design. Our results contribute a straightforward platform for instant AUT evaluation and provide valuable ground truth data for future methodological research.

## BACKGROUND

Creativity is defined as the ability to generate ideas that are both novel and useful (Runco & Jaeger, 2012). Novelty, also referred as originality (Amabile, 1983), involves exploring unique approaches to open‐ended problems (Guilford, 1950). On the other hand, usefulness corresponds to the real‐world applicability of an idea and is usually defined in terms of both feasibility and value (Litchfield et al., 2015). While feasibility refers to the practicality of an idea (Poetz & Schreier, 2012; Rietzschel et al., 2010), the concept of value lacks a universal definition. Some associate it with monetary worth (Lepak et al., 2007), whereas others argue that value should measure an idea's effectiveness in achieving goals, which may not always be financial (Ford & Gioia, 2000).

To evaluate creativity, divergent thinking tests are most commonly used. These tests, such as the popular Alternative Uses Test (AUT), prompt participants to generate ideas, often asking for unconventional uses for everyday items like umbrellas or bricks (Runco et al., 2016; Snyder et al., 2019). Research shows that these tests are strong predictors of individual creative ability and potential (Kim, 2008; Runco & Acar, 2012; Runco et al., 2010; Said‐Metwaly et al., 2022). Such tests of creativity are often scored on a Likert scale by a small group of judges, who are trained to use a consensual definition of creativity (Amabile, 1982). Scoring is often limited to the novelty or originality of responses, with other aspects of creativity overlooked. Studies argue that a comprehensive creativity assessment should measure both novelty and usefulness, not only because they play distinct roles in evaluating creativity (Diedrich et al., 2015), but also to improve the tests' validity in reflecting real‐world creativity (Zeng et al., 2011).

In addition to manual scoring by trained judges, automated scoring methods of divergent thinking tests have been developed. One such approach is to use latent semantic analysis (Landauer et al., 1998) and other text mining approaches, where words are considered in their context in a large text corpus and mapped into a high‐dimensional latent space, such that words that often appear together or share similar contexts are located close to each other in this space. Distance between words in this latent space, termed semantic distance, can be interpreted as a measure of conceptual dissimilarity. Semantic distance between prompt and response has been shown to correlate to human judgements of originality in the AUT (Beaty & Johnson, 2021; Dumas et al., 2021; Dumas & Dunbar, 2014), but is not obviously related to feasibility or value judgements. More recently, large language models (LLMs) have been investigated as tools to evaluate creativity. LLMs are neural networks trained on huge corpora to predict the next word in a text based on the surrounding context. Such models can be further trained with task‐specific data such as creativity test prompts, responses and the corresponding originality ratings, to generate models that predict human ratings on unseen responses (Luchini et al., 2023; Organisciak et al., 2023). Luchini et al. (2023) used this approach also to score the quality of responses in a long‐form divergent thinking task and showed that their fine‐tuned model generalizes to related tasks unseen during training. Finally, Organisciak et al. (2023) showed that one of the most powerful LLMs currently available, GPT‐4 (OpenAI, 2023), is capable of rating the originality of AUT responses without fine‐tuning when the context fed to the model includes human‐rated examples.

In this study, we use a large language model, GPT‐4 (OpenAI, 2023), to assess not only novelty, but also feasibility and value of responses in a Japanese‐language AUT. We explore the impacts of several different strategies of requesting ratings on rating quality. In particular, we vary whether ratings are requested in each dimension separately or in all dimensions at once and how many items are rated in one request, from a single item up to lists of 50 items. In requests to rate short lists on a single dimension, we further explore the impact of providing human‐rated example ratings as part of the request to the model and of prompting the model to explain its reasons for the ratings.

To obtain high‐quality multi‐item ground truth data, we use a crowdsourced evaluation method involving 64 human evaluators and the Elo rating system (Elo, 1986). Originally designed for ranking players in Chess and Go tournaments, the Elo system has proven effective in other quality‐rating contexts as well (Clark et al., 2018; Paulsgrove et al., 2021). The Elo rating approach, though not previously used in creativity research, has several advantages over the standard approach to manual scoring. In the typically used consensual assessment technique (CAT) (Amabile, 1982), judges rate items on a Likert scale, while adhering to a consensus definition of the dimension (e.g., originality) rated, scaling their ratings to match both the other judges involved and the overall quality of responses. This is cognitively demanding, particularly for a large dataset. In contrast, in an Elo rating system, judges need only decide which response of a pair scores higher on a given dimension, a much less demanding task. Thus, judges are less likely to become fatigued and can rate more items more reliably. Furthermore, since no coordination between judges is required, more judges can be used and larger datasets rated, with each judge only rating parts of the data. Finally, the resulting ratings are explicitly relative to the overall quality of responses and can be of higher resolution, since items with close ratings are contrasted to break ties.

Using Elo ratings of novelty, feasibility and value, we then evaluate various prompt designs using GPT‐4, considering both performance and cost. We find good performance from chain‐of‐thought prompting (Wei et al., 2023), where in addition to a score, the LLM is asked to provide its reasoning in the form of a brief statement before providing a numeric score. In contrast, we find little benefit to providing ratings from withheld portions of the ground truth data as part of the prompt to the model (i.e., as in‐context training).

## METHODS

### Alternative Uses Test (AUT)

The current study includes Japanese‐language responses to AUT prompts from two sets of experiments conducted with 67 native Japanese speakers (33 female, 34 male, age: mean ± standard deviation = 24.6 ± 3.7 years). Each participant performed 30 trials (i.e., 30 objects) of the AUT (Guilford, 1967). In each trial, participants were asked to come up with an uncommon alternative use for a given object with the following format, translated from Japanese: ‘Using 【item】as <alternative use (noun)> (e.g., using 【umbrella】as <a walking stick>)’. Responses (i.e., alternative uses) were given in Japanese. For evaluation purposes, we removed duplicate object/use pairs, which left us with 1710 responses, or an average of 57 (46 ~ 64) alternative uses for each object. Participants were recruited through a website (https://www.jikken‐baito.com). They all signed informed consent before participation and were provided with monetary compensation for their participation. All study protocols were approved by the Research Ethics Committee at The University of Tokyo.

### Subjective rating of AUT ideas

We rated the creativity of the proposed alternative uses with the following three dimensions: Novelty, Feasibility and Value. We adopted a crowdsourced evaluation approach that included 64 human evaluators (25 female, 39 male, age: mean ± standard deviation = 22.8 ± 2.3 years), who were recruited from the same website as AUT participants and provided with monetary compensation for their participation. Furthermore, instead of assigning subjective ratings for a given alternative use (e.g., Fink et al., 2007, 2006), the evaluators were asked to perform judgements in a two‐alternative forced‐choice manner, judging which of two alternative uses of a given object is greater in one of the three dimensions. This approach significantly reduces evaluators' burden of maintaining consistent rating criteria, as required in the Likert rating approach (Clark et al., 2018). Instructions were given to evaluators in Japanese; the English equivalents are given in Table 1. It is important to note that comparisons between alternative uses were performed only within the same object. In other words, for each object, all unique alternative uses were pooled together for the pairwise matches.

**TABLE 1:** Instructions for evaluators.

Dimension	Instruction
Novelty	Which『alternative use』of the given【item】is more unique, original or surprising? (disregarding feasibility and value)
Feasibility	Which『alternative use』of the given【item】is more practical or doable in real‐life settings? (disregarding novelty and value)
Value	Which『alternative use』of the given【item】could be sold for a higher price if both were equally feasible? (disregarding novelty and feasibility)

Participants were given 90 min to complete as many rounds of evaluation as possible. Each round consisted of one block of comparisons for each of the three dimensions, the order of which was chosen pseudo‐randomly for each round. A block consisted of 124 comparisons in the given dimension, including four comparisons for each of the 30 objects drawn from the AUT data, as well as four sanity check questions. The selection of the pair of alternative uses for a match was pseudo‐randomly assigned with higher priority to select two alternative uses with low total match numbers and closer ratings in the given dimension (see below). The sanity check questions were composed of a pair of alternative uses and designed to have obvious win‐loss judgement. For example, one sanity check question for Novelty is to compare ‘using umbrella as a fish tank’ with ‘using umbrella as a weapon’. In this case, the former should be considered more novel than the latter. The sanity check questions were included to prevent participants from picking randomly and/or to reduce the potential noise if a participant failed to understand the tasks. If a participant failed more than one of the four sanity check questions in a single block (e.g., Novelty), the data from that participant and block was discarded. Participants typically performed one to three rounds of evaluation within the allotted time. On average, each alternative use was compared 16.8 times (14 ~ 26 times) for novelty, 18.4 times (15 ~ 28 times) for feasibility and 13.6 times (10 ~ 21 times) for value.

The subjective ratings for alternative uses were determined by the Elo rating algorithm (Elo, 1986) based on their match results with other alternative uses. There are two primary characteristics of the Elo rating algorithm. First and most intuitively, a player with more wins will have a higher score. Second, score updates depend on not just the outcome of a match, but also on the score of the opponent, such that winning or losing against higher‐ranking opponents yields a greater rank reward or deduction, respectively, and vice versa for lower‐ranking opponents. In the current study, we treated alternative uses as players, using the Elo rating algorithm to obtain subjective ratings. The ratings of all alternative uses were updated after every round and the updated ratings were used to assign the matches for the next round for a human evaluator.

### The Elo rating algorithm for subjective ratings

The Elo rating scores were determined in two steps. First, for a given match of two alternative uses *A* and *B*, we calculated the expected odds of winning, which are denoted as *E*_*A*_ and *E*_*B*_ respectively, with the following equations:

(1) $$E_{A}=\frac{1}{1+10^{\left(R_{B}-R_{A}\;\right)/400}}$$

(2) $$E_{B}=\frac{1}{1+10^{\left(R_{A}-R_{B}\;\right)/400}}$$

Here, *R*_A_ and *R*_B_ denote the initial ranking scores of *A* and *B*, respectively, before entering the match. Based on *E*_A_ and *E*_B_ and the match result, we can then calculate the new ranking scores *R*′_A_ and *R*′_B_ as follows:

(3) $$R_{A}^{\prime }=R_{A}+K\;\left(S_{A}-E_{A}\right)$$

(4) $$R_{B}^{\prime }=R_{B}+K\;\left(S_{B}-E_{B}\right)$$

Here, *S*_A_ and *S*_B_ denote the match outcomes with 1 referring to a win and 0 referring to a loss. *K* denotes the *K*‐factor, a score adjusting constant, and is set to be 16 in the current rating system. Scores for all alternative uses were initialized to a score of 1600. By running the algorithm iteratively through all matches, we obtained corresponding Elo ratings for all alternative uses in Novelty, Feasibility and Value for a given object.

There is no clear consensus on how many matches are required for stable Elo ratings, since ratings approach their asymptotic value roughly exponentially. Other authors using the Elo method are satisfied with as little as nine matches (Neumann et al., 2011), to which our data with 10 to 28 matches per item compare favourably. We assessed stability with respect to the choice of *K*, which had virtually no impact on rank order, see Figure S13. We also assessed the stability of the resulting ratings with respect to the number and order of matches as follows. We shuffled the order of matches five times, concatenated these five sets of matches and calculated the Elo scores in the above manner. Then, we calculated the Spearman's rank correlation between these shuffled Elo scores and the scores obtained by a single pass in the original order. The resulting correlation coefficients were 0.98 for novelty, 0.98 for feasibility and 0.99 for value, indicating that the single‐pass scores were stable. For further analysis of stability with respect to match order, see Figure S14.

### Consent

All participants involved in this study were provided with a detailed informed consent form outlining the purpose, procedures, potential risks and their rights as participants and they voluntarily signed the form prior to their participation.

### Obtaining ratings with GPT‐4

We used OpenAI's GPT‐4 language model (OpenAI, 2023) to rate the alternative uses in the three dimensions of Novelty, Feasibility and Value. Briefly, GPT‐4 is a large language model (LLM) trained on huge amounts of natural language data, both in English and in other languages. The model takes inputs in the form of so‐called prompts and produces text or text‐like output in response. While GPT‐4 is proprietary and closed‐source, OpenAI provides access through an API, i.e., through a pay‐per‐use interface that allows external software such as our code to send queries and receive responses computed by the model.

### Prompting approaches

In problem‐solving domains, it has been shown that LLMs like GPT‐4 can be much more reliable when prompted to elaborate on their solutions before committing to an answer, an approach known as chain‐of‐thought prompting (Wei et al., 2023). We therefore adopted a similar strategy, prompting not just for a numeric rating, but for a natural language elaboration related to the requested rating. We were careful to clearly delineate in our prompts both the dimensions under consideration, i.e., the model's task description and the format of the expected outputs, adding as much detail as necessary to obtain rich, but easily processed responses. In an attempt to increase the resolution of the ratings obtained, we prompted for ratings from 1 to 100 for Novelty and Feasibility. For Value, we requested an estimate of the market price in JPY of the alternative use (Lepak et al., 2007). Representative sample prompts illustrating prompt structure and the definitions of the three dimensions are presented in Table 2.

**TABLE 2:** Prompt building blocks.

Building block	Approaches	Prompt
Introduction	1 dimension (see below for the definitions of Feasibility and Value)	We aim to evaluate the creativity of ideas in an Alternative Uses Tests (AUT) based on specific criteria. Please rate our ideas, given below, in terms of their Novelty, which is defined as follows: Novelty: Rate the novelty of the idea on a scale of 1 to 100, with 1 being not novel at all and 100 being extremely novel. Consider how unique, original, or surprising the idea is, while disregarding its feasibility and value.
	3 dimensions	We aim to evaluate the creativity of ideas in an Alternative Uses Tests (AUT) based on specific criteria. Please rate our idea, given below, in terms of its Novelty, Feasibility, and Value, which are defined as follows: Novelty: Rate the novelty of the idea on a scale of 1 to 100, with 1 being not novel at all and 100 being extremely novel. Consider how unique, original, or surprising the idea is, while disregarding its feasibility and value. Feasibility: Rate the feasibility of the idea on a scale of 1 to 100, with 1 being not feasible at all and 100 being extremely feasible. Consider how practical or doable the idea is in real‐life settings, while disregarding its novelty and value. Value: Estimate the potential commercial value of the idea in Japanese yen, while disregarding its novelty and feasibility.
Procedure	3 dimensions, elaborated	Proceed as follows in your evaluation. First, briefly describe the idea in your own words. Then, in a short paragraph, consider a few other unconventional uses of the item, and compare the idea to these in terms of its Novelty. In a second paragraph, consider the Feasibility of our idea, outlining possible challenges to implementation. In a third paragraph, consider existing products achieving the same purpose as our idea, and estimate their value. Finally, provide your numeric ratings as a json object of the form {“novelty”:x, “feasibility”:y, “value”:z}, where z is an integer representing the value in JPY.
	1 dimension, elaborated	Proceed as follows in your evaluation. Write 3 (three) lines for each item in the list below. On the first line, write the item number and briefly describe the idea in your own words. On the second line, consider other unconventional uses of the item, including those listed, and compare the idea to these in terms of its Novelty. Finally, on the third line, provide your numeric rating as a json object of the form {“novelty”:x}. Evaluate each idea in the order provided, leaving one empty line between evaluations. Do evaluate each of the 20 ideas below individually, even if there are repetitions.
	Rating only	Provide your ratings as a numbered list with one item per line, in purely numeric format. Do evaluate each of the 20 ideas below individually, even if there are repetitions.
Samples	With samples	The following examples illustrate the expected range of final Novelty ratings for alternative uses of the item “ボールペン”: Example 1: 棒 ‐‐ 37 *[…]*
	Without samples	*[omitted]*
Answer(s)	One answer	Our idea is to use the item “ボールペン” as “冷蔵庫”.
	Multiple answers	Our ideas are to use the item “ボールペン” in the following ways: 1. 冷蔵庫 *[…]*
System	(all)	You are an advanced instruction‐following AI agent. You are highly trained in English and Japanese and intricately aware of Japanese culture. Your responses summarize the average opinions of the Japanese general public.

*Note*: The order (as presented in the table) and formulation of the building blocks was conserved across all approaches, with necessary adjustments for the number of alternative uses and dimensions requested. The system message, designed to shape the high‐level model behaviour, was inserted between the end of the prompt and the model response. Portions in italics and hyphenation are supplied for the benefit of the reader and were not included in the prompts. Building blocks were separated by two newline characters.

**FIGURE 1:**
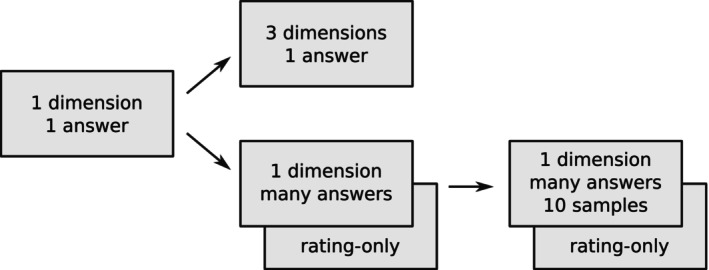
The various approaches taken to prompt GPT‐4 for ratings in the dimensions of Novelty, Feasibility and Value. Unless noted otherwise, an elaboration was requested ahead of the numeric rating.

To explore the capabilities and limitations of obtaining ratings with GPT‐4, we investigated several different approaches, as illustrated in Figure 1. Besides a naïve approach, where each prompt yields a rating in one dimension of one answer (i.e., one object/use pair), we used a combined prompt requesting all three ratings in one response and list prompts asking for ratings in one dimension for several alternative uses of a single object, both with and without in‐context learning samples. We tested list prompts consisting of 10, 20 and 50 answers to rate. When including training samples in what is termed in‐context learning in the AI field, we always used 10 samples for a given object/dimension combination, providing the human‐rated scores of the queried dimension only.

### Training samples

Training samples were obtained from the Elo rated data as follows. For each object and each dimension, we chose 10 alternative uses approximately covering the full range of Elo ratings while maximizing the overlap between choices across dimensions. Briefly, responses were sorted by Novelty, separated into 10 bins and the median item of each bin selected as a sample for Novelty. Then, all responses were sorted by Feasibility and separated into 10 bins. In each bin, if no response had yet been chosen as a sample in either dimension, the median was selected; otherwise, of the responses already chosen as samples, the one closest to the bin median was selected as a sample for Feasibility. This procedure was repeated analogously for Value and served to reduce the number of responses excluded from evaluation (due to being used as samples) from at most 900 (30 objects × 3 dimensions × 10 samples) to just 488. The chosen samples were excluded from the dataset, resulting in the remaining *n* = 1222 answers being available for evaluation and analysis. Sample Elo ratings were linearly converted to a scale of 1 to 100 using the minimum and maximum Elo ratings across the entire dataset. Finally, all 10 samples of the appropriate object and dimension were added to the list prompts in ascending order alongside the converted rating, but without any elaboration.

### Technical details

We used the latest version of GPT‐4 at the time of writing, version gpt‐4‐0613, and a temperature setting of 0 along with default settings for other parameters (top_p = 0, presence_penalty = 0, frequency_penalty = 0). Despite our best efforts, responses occasionally failed to follow the requested format or rate all given answers, in which case the request was repeated with a temperature increment of 0.1. In no case did we observe more than three consequent failures of the same prompt.

### Data analysis

Model responses that followed the requested format were converted into numerical ratings for analysis. Data were analysed without any sanitization efforts in the Novelty and Feasibility dimensions, which were consistently within the requested range of 1–100. In contrast, Value ratings occasionally yielded extremely large or negative values. We treated these as outliers, removing any data points with values less than zero or greater than 100,000 (JPY, corresponding to approximately 700 USD) for further analysis.

To estimate the temporal and financial costs associated with each prompt strategy, we pseudo‐randomly sampled 25 requests (i.e., complete prompts requesting ratings of either one answer or a list of answers, depending on the approach) from our dataset. We sent the requests one at a time, tracking their round‐trip latency and the number of prompt and response tokens consumed. We estimated the financial cost based on OpenAI's per‐token pricing model for GPT‐4, which, at the time of writing, was set at $0.03/1000 prompt tokens and $0.06/1000 response tokens. We then averaged both time and cost across all requests to arrive at a value per individual rating.

### Benchmarking

We obtained four publicly available English‐language datasets of AUT responses (Beaty et al., 2018 [betal18]; Beaty & Silvia, 2012 [bs12]; Hofelich Mohr et al., 2016 [hmsl]; Silvia et al., 2017 [snb17]) from https://conservancy.umn.edu/handle/11299/172116 (hmsl) and https://osf.io/gz4fc/ (bs12, snb17, betal18). Within each dataset, we removed duplicate object/use pairs, then rated their Novelty with the 1 dimension, 20 answers approach without training samples. Results in the form of Pearson correlation coefficients between GPT‐driven and human‐judged ratings were compared to those reported in (Organisciak et al., 2023) for a degree of independent verification of our approach.

## RESULTS

### Qualitative observations

Sample responses for a representative AUT object/use pair are given in Table 3. We confirmed in spot checks across the full set of responses that the Japanese items and alternative uses were generally translated and interpreted appropriately. Responses in the single answer approaches typically yielded a large amount of text output with detailed and usually reasonable elaborations, making the requested comparisons (Novelty), discussing barriers to implementation (Feasibility) and judging the potential Value fairly, though often – despite instructions to ignore the other dimensions – with reference to the feasibility of the proposed use.

**TABLE 3:** Sample responses.

Approach	Response
3 dimensions 1 answer	The idea is to use a ballpoint pen (“ボールペン”) as a refrigerator (“冷蔵庫”). In terms of novelty, this idea is quite unique. Ballpoint pens are typically used for writing or drawing, and the concept of using one as a refrigerator is certainly out of the ordinary. Other unconventional uses for a ballpoint pen might include using it as a makeshift hairpin, a tool for pressing small buttons, or a simple toy. Compared to these, the idea of using a ballpoint pen as a refrigerator is significantly more novel. However, the feasibility of this idea is extremely low. A ballpoint pen is a small, handheld item that lacks the necessary components to function as a refrigerator. It does not have the capacity to store items, nor does it have a cooling system. Implementing this idea would require a complete redesign of the ballpoint pen, essentially turning it into a different product altogether. In terms of value, a typical refrigerator in Japan can range from 30,000 to 300,000 JPY, depending on the size and features. However, given the impracticality of this idea, it is unlikely that a ballpoint pen refrigerator would have any commercial value. Even if it were possible to create such a product, it would likely be seen as a novelty item rather than a practical appliance. {“novelty”: 90, “feasibility”: 1, “value”: 0}
1 dimension 1 answer	The idea is to use a ballpoint pen (“ボールペン”) as a refrigerator (“冷蔵庫”). In comparison to other unconventional uses of a ballpoint pen, such as using it as a hairpin, a makeshift ruler, or a tool to reset electronic devices, the idea of using it as a refrigerator is highly unique. It's not immediately clear how a pen could function as a refrigerator, which adds to the novelty of the idea. However, it's worth noting that the novelty of an idea doesn't necessarily correlate with its feasibility or practicality. {“novelty”: 95}
1 dimension 20 answers Novelty	1. Idea: Using a ballpoint pen as a refrigerator. This idea is extremely novel, as it's hard to imagine how a pen could function as a refrigerator. It's a very unique and surprising use. {“novelty”: 100} *[…]*
1 dimension 20 answers Feasibility	1. Idea: Use a ballpoint pen as a refrigerator. Feasibility: This is not feasible as a ballpoint pen lacks the necessary components and size to function as a refrigerator. {“feasibility”: 1} *[…]*
1 dimension 20 answers Value	1. Refrigerator The value of a refrigerator is quite high, ranging from 30,000 to 200,000 JPY. {“value”: 115000} *[…]*

In contrast, list prompts yielded brief elaborations with a minimum of detail, often ignoring the instruction to compare to other uses (Novelty) or failing to make specific statements about barriers to implementation (Feasibility). In the Value dimension, the list prompt responses typically simply stated the price range of the use by way of elaboration and offered the midpoint of that range as the rating.

**FIGURE 2:**
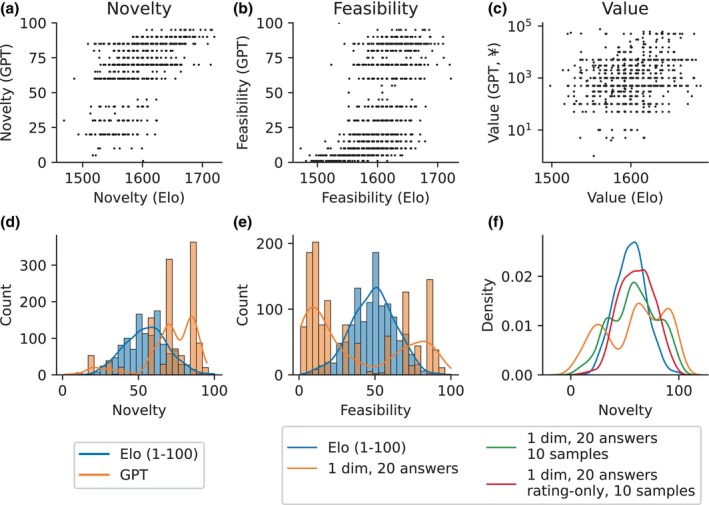
Comparison of Elo ratings with GPT‐driven ratings using the 1 dimension, 1 answer approach. (a–c) Scatterplots directly comparing ratings. (d, e) Rating distributions (histograms and Gaussian kernel density estimation) in the Novelty and Feasibility dimensions. For the purposes of these distributions, Elo ratings were linearly converted to a 1–100 scale using the minimum and maximum Elo ratings in the respective dimension across the entire dataset. (f) Novelty rating distributions across several list prompts, displayed by way of kernel density estimations.

In the following, we will ignore the elaborations and focus exclusively on the numeric evaluations and their relation to the Elo ratings, which we treat as a ground truth measure. In Figure 2a–c, we show the relationship between Elo ratings and GPT ratings from the one dimension, one answer approach. The corresponding plots for all other approaches are supplied in Figures [Link], [Link], [Link], [Link], [Link], [Link], [Link]. In the Novelty and Feasibility dimensions, we see a clearly positive relationship vaguely resembling a logistic function, as one might expect from plotting a uniform distribution (GPT ratings, 1–100) against a normal distribution (Elo ratings, centered on the starting value of 1600). In the Value dimension, the relationship is less clear. Since we requested a commercial value from GPT‐4, the returned values span several orders of magnitude, rather than being restricted in a fixed range like the other dimensions. As a result, the mapping between GPT and the Elo ratings takes a different shape, though the relationship remains clearly positive across all items. (Note, for a quantitative analysis of the correlations on a per‐question basis, please refer to the next section).

Considering the distribution of ratings (Figure 2d,e), we see that, while Elo ratings are approximately normally distributed around the initial value of 1600, the GPT ratings follow a different pattern, with many very high ratings of Novelty and an approximately bimodal distribution in Feasibility, reflecting roughly binary judgements, with most alternative uses judged either largely feasible or largely impossible.

Qualitatively, other approaches yielded similar results, with two notable exceptions, both in the Novelty dimension. Firstly, in list prompts, the distribution of Novelty ratings was more balanced, suggesting that the inclusion of multiple alternative uses allowed GPT‐4 to make meaningful comparisons. Notice, however, that such comparisons appear to have been made implicitly, since list prompt responses, as noted above, did generally not include any explicit comparisons in the elaboration. Secondly, both the inclusion of samples and the reduction to simply returning numeric ratings appeared to lead to a closer approximation of the distribution of Elo ratings than the list prompt without samples, but with elaboration.

To confirm this numerically, we first converted the Elo ratings to a 1–100 scale (in the same manner as for samples, see Methods for details), then estimated the probability densities of the Novelty ratings by Gaussian kernel estimation, shown in Figure 2f. Next, we calculated the Kullback–Leibler divergence (*D*_KL_) between probability densities of the Elo and GPT‐driven ratings. Briefly, *D*_KL_ measures the difference between two distributions, with *D*_KL_ = 0 indicating identity and larger values indicating greater divergence (Kullback & Leibler, 1951). We found that, indeed, the distributions in the 20‐answer prompt with elaboration were least similar (*D*_KL_ = 0.54) and both adding samples (*D*_KL_ = 0.19) and removing elaborations (*D*_KL_ = 0.26) individually led to greater distributional similarity. Finally, the greatest similarity between Elo and GPT‐driven ratings was achieved by a rating‐only prompt with samples (*D*_KL_ = 0.09).

A similar trend was observed for Feasibility (see Figure S8), though it was both visually and numerically less striking, with *D*_KL_ varying between 0.8 for the basic elaborated list prompt and 0.43 for the approach with samples and no elaboration.

### Comparison between approaches

Greater distributional similarity, however, does not necessarily reflect higher quality ratings. To rigorously compare our chosen approaches, we calculated the Spearman correlation *ρ* between the Elo and GPT‐driven ratings, separating by item and dimension and compared these correlations. For brevity, we will refer to GPT‐driven ratings that correlate more strongly with the Elo ratings as ‘more accurate’, despite possible concerns in evoking notions of accuracy in what may be considered a subjective assessment.

First, comparing the single answer prompts with only one or all three dimensions, we might expect that the evaluation in one dimension might bias the evaluation in another in the context of three‐dimensional prompt, in which case we should see higher accuracy in the one‐dimensional prompt. Conversely, the one‐dimensional prompt only defined the requested dimension, yet asked the model to disregard the other, not clearly defined, dimensions. Consequently, we might expect the one‐dimensional prompt to underperform due to these unclear instructions. In fact, consistent with the former hypothesis, we found (Table 4) that the one‐dimensional prompt yielded more accurate ratings in both Novelty and Feasibility, suggesting that, indeed, requesting several unrelated judgements on the same object/use pair degraded performance. In the Value dimension, we found that the correlations barely differed between the two approaches.

**TABLE 4:** Spearman correlations between Elo ratings and GPT‐driven ratings.

Prompt method	Novelty			Feasibility			Value		
	*M*	std	SNR	*M*	std	SNR	*M*	std	SNR
3 dims, 1 answer	0.51	0.14	3.64	0.57	0.12	4.66	0.25	**0.13**	1.92
1 dim, 1 answer	0.55	0.13	4.35	0.67	**0.10**	**6.43**	0.24	0.18	1.36
1 dim, 10 answers	0.61	0.12	5.03	0.62	0.14	4.30	0.33	0.18	1.83
1 dim, 20 answers	0.64	**0.10**	**6.24**	0.60	0.14	4.41	0.36	0.16	2.29
1 dim, 50 answers	**0.65**	0.11	5.80	0.62	0.13	4.83	**0.43**	0.14	**3.05**
1 dim, 20 answers, 10 samples	0.63	0.12	5.17	0.62	0.11	5.58	0.39	**0.13**	2.95
1 dim, 20 answers, rating‐only	0.38	0.25	1.53	0.65	0.12	5.32	0.39	0.17	2.26
1 dim, 20 answers, rating‐only, 10 samples	0.52	0.25	2.04	**0.68**	0.13	5.29	0.31	0.22	1.40

*Note*: Correlations are calculated per item and presented as mean (M) and standard deviation (std) across items. The third column, SNR, represents the signal‐to‐noise ratio, calculated as *M*/std. The best values in each column are highlighted in bold. For the distributions of correlations separated by prompt method and dimension, see Figures [Link], [Link], [Link].

Next, we examined the impact of rating multiple answers in a single prompt. Here, we might expect to see improvements in the accuracy of Novelty ratings the more answers are presented simultaneously, particularly because our ground truth data, the Elo ratings, are explicitly comparative and more generally because the Novelty of a creative idea is necessarily judged in relation to other ideas. In contrast, we might expect Feasibility ratings to be more absolute and thus less affected by comparisons between answers, though there may be effects of calibrating the interpretation of the 1–100 rating scale. Finally, we would expect the least effect in the Value domain, which we asked GPT‐4 to judge on absolute terms. However, note that for both Feasibility and Value ratings, the ground truth data were also comparative, such that giving GPT‐4 more answers to compare may improve consistency with Elo ratings, if to a lesser degree than for Novelty.

As shown in Table 4, we indeed found that offering multiple answers for comparison improved accuracy for Novelty. Accuracy improvements levelled off quickly, which suggests that the effect of comparability had a very low ceiling in our dataset. For Feasibility, we found the highest correlations in the single‐answer prompt, suggesting that Feasibility was judged largely on a per‐answer basis, with possibly some ‘contamination’ when multiple answers were presented. Finally, we found a clear increase in accuracy with increasing answer numbers in the Value dimension, showing higher variability, but a similar saturation trend as the correlations in Novelty. We suspect that the lower accuracy in the single‐answer prompts may be caused by the longer elaboration, which often led the model to conclusions that included not only market price, but also, despite the instructions, feasibility considerations.

Thirdly, we considered the effect of in‐context learning, focusing on a 20‐answer list prompt approach. We might expect that providing some rating examples would allow the model to calibrate its outputs, improving correlations in Novelty and Feasibility. In fact, as shown in Table 4, the effect of providing samples was very limited. For Novelty, the correlations slightly degraded with the addition of 10 samples, while the correlations for Feasibility slightly improved. The absence of a noticeable effect suggests that either the response was not strongly influenced by the samples (but see the shift in rating distributions discussed above), or the model outputs already resembled the Elo ratings well enough that the samples were unable to improve accuracy.

For Value, the situation was a little different. The samples were presented with ratings from 1–100, yet the prompt was otherwise unchanged, asking for a valuation in JPY. We might expect that this incongruity would confuse the model and degrade performance. Instead, we found that the model (correctly, from the point of view of the request) correctly insisted on providing valuations in JPY with only minor changes from the prompt without samples. Yet, the average correlation increased slightly. This lends further support to the hypothesis that the samples were largely ignored.

Finally, we considered the impact of the requested elaboration in the 20‐answer list prompts. Our expectation was that the elaboration would allow the model to firmly ground its rating response with a preceding rationale and thus, without such elaboration, we expected to see a clear drop in performance in both Novelty and Feasibility, while the more objective Value estimate should be less affected. Indeed, we found that, when the response was limited to ratings, Novelty ratings were considerably less accurate and more variable, leading to lower signal‐to‐noise ratios (Table 4). Providing samples partly rescued accuracy without improving variability. In contrast, Feasibility ratings substantially improved when elaborations were removed and improved further with the addition of samples, while variability remained at comparable levels. Finally, Value judgements were slightly improved in accuracy by the removal of elaborations, while the additional introduction of samples reduced accuracy and increased variability, likely reflecting confusion over the conflict between the request (valuation in JPY) and the samples (valuations from 1 to 100).

### Cost considerations

**FIGURE 3:**
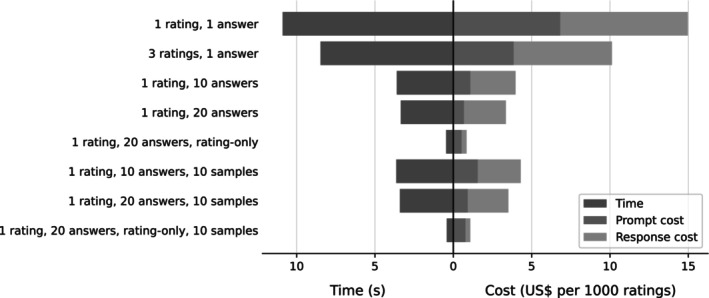
Analysis of cost per answer and rating across all approaches, estimated from 25 API requests per approach. Left: Time between request being sent and full response received. Right: Cost under OpenAI's pricing per token, broken down by tokens in the input to and output from the model. Note that pricing is model‐specific and may change; the values shown here reflect pricing for the gpt‐4‐0613 model ($30/60 per million input/output tokens, respectively).

Since each request to the OpenAI API takes some time to complete and is charged based on the number of ‘tokens’ (typically short words or word chunks), rating large datasets can become prohibitively expensive or long to run with an inappropriate prompting strategy. We therefore investigated the costs associated with the prompts used in this study, summarized in Figure 3.

We found that the single‐answer prompts were substantially more costly than the list prompts, since each request had to include the introduction, dimension definitions and instructions for how to respond. In contrast, the impact of list length (10 or 20 answers) and of samples was small enough to be negligible. A further steep reduction in price and time required could be achieved by omitting the request to elaborate, as this reduced the output to a simple list of numbers.

### Model comparison

During the revision of this paper, several new language models were released, allowing us to compare performance between these. GPT‐4 was updated, first to Turbo (gpt‐4‐0125‐preview), then to Omni (gpt‐4o‐2024‐05‐13) and an OpenAI competitor, Anthropic, released Claude 3.5 (claude‐3‐5‐sonnet‐20240620), which performs similarly to Omni in generic benchmarks (Anthropic, 2024; Huang et al., 2024). We tested our list prompts in all three of these newer models to understand to what extent the results noted above were specific to the model and version (gpt‐4‐0613) used. The single‐answer prompts were not tested out of cost considerations.

Since Claude consistently produced output in Japanese instead of the expected English, we added the sentence ‘You reply exclusively in English.’ to the system message. This addition was used for Claude, but also for Omni and Turbo, and served to ensure English output throughout. All models generally followed the instructions well without adjustment to the prompts, though idiosyncrasies in the output formatting had to be accounted for to extract ratings. Generally, Claude appeared to follow formatting instructions more closely than the various versions of GPT‐4.

Results are summarized in Table 5. Of the four models, Omni tended to yield the best accuracy with respect to the Elo ratings in most prompt approaches. While there were some differences between models, the overall performances were similar and the trends observed above for GPT‐4 largely held; neither the newer GPT versions nor Claude consistently benefited from samples in the prompt and all models lost accuracy (Novelty, Value) or gained accuracy (Feasibility) when text elaborations were omitted.

**TABLE 5:** Means and signal‐to‐noise ratios of Spearman correlations between Elo ratings and LLM‐driven ratings, using models GPT‐4 (gpt‐4‐0613), Turbo (gpt‐4‐0125‐preview), Omni (gpt‐4o‐2024‐05‐13) and Claude 3.5 Sonnet (claude‐3‐5‐sonnet‐20240620).

Method	Novelty				Feasibility				Value			
Model	GPT‐4	Turbo	Omni	Claude	GPT‐4	Turbo	Omni	Claude	GPT‐4	Turbo	Omni	Claude
1 dim, 10 answers	0.61	0.62	0.64	0.59	0.62	0.59	0.63	0.57	0.33	0.26	0.37	0.36
	5.03	4.95	**6.24**	5.54	4.30	4.15	5.12	4.41	1.83	1.41	2.30	2.46
1 dim, 20 answers	0.64	0.60	0.64	0.58	0.60	0.59	0.66	0.59	0.36	0.26	0.38	0.36
	**6.24**	4.10	5.24	3.71	4.41	3.83	6.28	3.58	2.29	1.48	2.21	2.00
1 dim, 50 answers	0.65	0.61	**0.66**	0.62	0.62	0.61	0.67	0.60	0.43	0.30	**0.47**	0.43
	5.80	4.32	5.48	4.26	4.83	3.87	**7.05**	4.61	**3.05**	1.51	2.54	2.44
1 dim, 20 answers, 10 samples	0.63	0.60	0.63	0.61	0.62	0.63	0.67	0.61	0.39	0.21	0.33	0.37
	5.17	4.32	5.32	4.56	5.58	4.77	6.62	4.08	2.95	0.92	1.71	2.06
1 dim, 20 answers, rating‐only	0.38	0.07	0.24	0.37	0.65	0.67	0.69	0.64	0.39	0.24	0.25	0.45
	1.53	0.17	1.04	1.48	5.32	5.89	6.38	5.79	2.26	0.89	1.19	2.81
1 dim, 20 answers, rating‐only, 10 samples	0.52	0.34	0.40	0.54	0.68	0.69	**0.71**	0.65	0.31	0.29	0.39	0.40
	2.04	1.25	1.85	2.85	5.29	5.58	5.51	4.75	1.40	1.49	2.37	2.52

*Note*: Correlations are calculated per item and presented as the mean (top row) and signal‐to‐noise ratio (mean/std, bottom row) across items. The best values in each dimension are printed in bold and all values within 90% of the maximum are highlighted in colour (blue for means, red for signal‐to‐noise ratios).

### Benchmarking

As shown in Table 4 above, our approach of requesting elaborations greatly improved the correlation between Elo ratings of Novelty and the corresponding GPT‐driven ratings. We therefore wondered how this strategy would fare in other datasets. We focused our investigation on the approach that had given us the best Novelty ratings, prompting for 20 answers at once without providing any samples. We targeted four English‐language datasets (Beaty et al., 2018 [betal18]; Beaty & Silvia, 2012 [bs12]; Hofelich Mohr et al., 2016 [hmsl]; Silvia et al., 2017 [snb17]) with a total of 8720 object/use pairs across four objects (brick, box, rope and paperclip) rated for ‘creativity’ or ‘originality’ on a 1–5 Likert scale by three to four human judges. We considered the mean rating across judges as the ground truth data.

**FIGURE 4:**
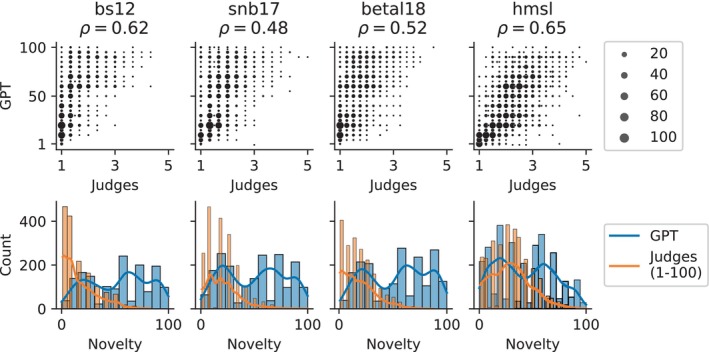
Comparison of human ratings of originality with GPT‐driven ratings of Novelty in four external datasets, using the 1 dimension, 20 answers approach. Top: Scatterplots directly comparing ratings. Marker size indicates the number of coincident points. Bottom: Rating distributions, shown as histograms and kernel estimations. For the purposes of plotting these distributions, human‐judged ratings were multiplied 20‐fold for alignment with the GPT‐driven ratings.

We first compared our results to the judges’ ratings as shown in Figure 4. We found a robust relationship between the two values in all four datasets, with Spearman correlation values in the same range as for our own data. There was a strong trend for GPT‐driven ratings to be higher than the corresponding ground truth data. The distribution of ratings, which was heavily skewed towards the low end of the scale in the human‐judged ratings, was considerably wider in GPT‐driven ratings, exhibiting a slight trend towards bimodality.

We then compared our results to those of Organisciak and colleagues (Organisciak et al., 2023), who used a minimal prompt, likewise with GPT‐4, to obtain creativity ratings for the same datasets. Since the authors only provide Pearson correlation coefficients, we calculated these for our ratings as well. The results are shown in Table 6. We find that while accuracy is roughly in the same range, our method performs better in only one of the four datasets.

**TABLE 6:** Pearson's correlation coefficients of GPT‐4 zero‐shot approaches with human judge ratings.

	bs12	snb17	betal18	hmsl
(Organisciak et al., 2023)	0.52	**0.52**	**0.57**	**0.64**
Ours	**0.55**	0.47	0.50	0.63

*Note*: The highest correlations in each dataset are highlighted in bold.

## DISCUSSION

In this study, we have demonstrated the use of GPT‐4 to assess the creativity of AUT answers in multiple dimensions. We showed that the results from this method align well with the relative ratings given by an ensemble of human judges. We tested the impact of several variations in prompt design and found roughly comparable performance across a wide range of approaches. Furthermore, harnessing the fact that GPT‐4 was trained on a multilingual corpus, we have demonstrated that it is possible to perform such ratings on Japanese data with minimal additional effort over English data and expect this to generalize to many more languages.

Counterintuitively, and in contrast to related work (Brown et al., 2020; Organisciak et al., 2023), we found little improvement from in‐context learning. This is particularly surprising given that the distribution of ratings does become more similar (Figure 2), but clearly corroborated by a corresponding lack of improvement in terms of goodness of fit (Figure S12). We can speculate that GPT‐4 may have used the samples simply as a baseline for the range and distribution of ratings, rather than to tune the mapping between AUT responses and their score in its evaluations. This failure to improve could be either due to the prompt, or to how the samples are presented. On the one hand, considering the prompt, there is no systematic way of tuning a natural language prompt and a search for a suitable formulation across the vast space of possible alternatives is expensive and certain to be incomplete. Additionally, research on models up to the size of GPT‐3 indicates that using a small amount of labelled data (e.g., alternative uses with ratings) for prompt selection becomes challenging as the model size increases (Perez et al., 2021). In other words, there is no guarantee that a prompt that performs particularly well on a subset of our data (or indeed on all of it) generalizes any better to unseen data than competing prompts. Thus, while there are many obvious modifications we could make to the prompt, some of which might improve accuracy on our data, we consider it unlikely that these would constitute decisive improvements in the general case, i.e., for other datasets. On the other hand, considering the sample presentation, we note that unlike the requested response, we did not include any elaboration in our samples. Thus, not only did they not provide a good model response, but they also failed to build a rationale for the ratings that GPT‐4 could have extrapolated for its own responses. It has been shown in other contexts that well‐chosen explanations of the expected problem‐solving strategy could substantially improve model performance (Lampinen et al., 2022). Including elaborations in our samples would constitute such an explanation and likely would have improved the quality of responses.

Our approach to prompting GPT‐4, which includes a request to elaborate the evaluation before giving a numeric rating, mirrors what is known as chain of thought prompting in problem solving domains (Kojima et al., 2023; Nye et al., 2021; Wei et al., 2023), which allows the model output to emulate a chain of reasoning that guides the model towards good answers. In our analysis, elaboration clearly improved the evaluation of Novelty, confirming our intuition that this evaluation is non‐trivial and benefits from generating additional context on the ‘scratchpad’ (Nye et al., 2021) of intermediate output. In contrast, the accuracy of Feasibility and, to a lesser degree, of Value judgements was reduced by elaboration, though these judgements also became less variable. For Feasibility, we suspect that the inclusion of an elaboration may have biased the final judgement. Elaborations could push the model to emphasize only a few challenges in implementation, making the assessment focus on whether these specific challenges can be addressed instead of evaluating the overall feasibility of the idea.

For Value, we have largely failed to make a strong case for evaluation with GPT‐4. We believe there are several good reasons for this failure, all of which are directly related to our operational definition of Value in commercial terms. Firstly, this definition made a direct comparison to the ground truth data, the Elo ratings, less obvious. However, notice that this should not affect the rank order correlation measure that we used to assess response quality. Secondly, we chose to request a Value judgement in the local currency. This may have been challenging for GPT‐4 for the simple reason that its training data may not include enough information on this subject. Conversely, a judgement in US dollars would have clashed with the system instruction to judge ‘as a member of the Japanese public’, i.e., on the same terms as our human judges and might have introduced market‐specific biases. Thirdly and most importantly, we believe that our difficulties in judging Value are largely due to the lack of a clear definition that makes sense while remaining orthogonal to the other dimensions. We attempted to disentangle Value from Feasibility by requesting a valuation of the alternative use alone, rather than of the combination of item and alternative use; however, this could yield perversely high values for fanciful ideas. Conversely, judging the commercial value of an item/use combination while disregarding Feasibility is often impossible. Finally, many alternative uses are not comparable to commercially available goods or services, defying attempts to evaluate them on such terms. Thus, while we believe that the value of a creative idea should be part of its evaluation, we have yet to find a convincing definition that works across all possible items and answers in the context of the AUT.

In comparing our approach on separate, English‐language datasets to the results by Organisciak et al. (2023), who also used GPT‐4, we found roughly comparable results under a zero‐shot (i.e., no examples) regime, even though only our prompt asked for elaborations. In our estimation, there are at least two factors that could help explain this lack of improvement. Firstly, the small number of judges rating each dataset (three to four judges per answer) may have led to biases in the ground truth data. These biases might not be reflected in the ratings of GPT‐4, making direct comparisons less insightful and setting a relatively low ceiling on the achievable accuracy. Secondly, we note that both the instructions for judges and the instructions for GPT‐4 in the prompt employed by Organisciak et al. (2023) use the terms ‘creativity’ and ‘originality’ interchangeably and without explicit definition. In contrast, our instructions (both to human judges in our own data and to GPT‐4) ask for ratings of ‘novelty’ with an explicit definition of the term excluding other dimensions of creativity. In future work, it might be of interest to obtain GPT‐driven ratings along all relevant dimensions of creativity and combine these into a single ‘creativity’ evaluation, which we would expect to better align with the equally broad human judgements. Organisciak et al. (2023) also provide a version of GPT‐3 fine‐tuned for creativity ratings. It would have been of great interest to us to compare their approach of fine‐tuning and our approach of prompt engineering in our own dataset. However, while there is some multilingual capability in GPT‐3, its capacity of processing Japanese input is rather limited, such that no fair comparison could have been made.

Precisely because our dataset is in Japanese, however, we believe that other researchers may find it useful. Compared to existing datasets of AUT answers, our data are rated by more judges, with more than 10 judgements per answer. While these judgements did not directly yield an absolute evaluation, our use of the Elo rating system allows judges to evaluate answers relative to each other while maintaining a low cognitive burden, resulting in highly consistent ratings (Clark et al., 2018). Furthermore, with 30 items judged in the three dimensions of Novelty, Feasibility and Value, our dataset is very broad, providing a useful testing ground for other methods of evaluating creativity. Therefore, we have made it available online at https://osf.io/gdy6c/. Our experience with the AUT shows that short answers are often difficult to interpret. In future work, we hope to encourage participants to answer with short sentences instead, which would help to avoid ambiguity while eliminating any possible bias towards answers that can be captured in a single word.

A major limitation of our study is its reliance on a proprietary, closed source LLM. The model version we used, gpt‐4‐0613, may become inaccessible at any point, since more recent versions of GPT‐4 (Turbo and Omni) have become available. During revision of this manuscript, we had a chance to try these newer versions, along with a similarly powerful model from Anthropic and, as shown above, found only minor differences in performance between all of these models. However, since different models (and model versions) behave differently, it is entirely possible that prompts or approaches do not transfer well between them. In our case, only a few adjustments had to be made, both to the system message (to adjust the output language) and to the output parsing schemes (to extract the provided ratings). Despite the apparent robustness of our method in the face of different models, readers considering adopting a similar approach to ours should be aware that the world of large language models does not provide much solid ground at this time and applications of our method should be considered with an understanding of its preliminary nature.

Finally, we would be amiss not to mention our use of Elo ratings as the ground truth data, which is itself a departure from the usual approach to creativity assessment. While we believe that the advantages of our approach over the CAT (Amabile, 1982) – lower coordination requirements, greatly reduced risk of drifting evaluation criteria and reduced cognitive load for judges, among others – justify the use of Elo ratings, we acknowledge that it is an untested approach that requires validation against more standard assessment methods. We hope that our work inspires further investigations into the use of Elo ratings or similar ensemble rating approaches in this field.

To conclude, we believe that requesting ratings from large language models is a viable method of evaluating creativity which benefits from a certain objectivity, or at least repeatability, that is unavailable when using human judgements. With recent developments, we anticipate future work using not just language, but also the visual modality of GPT‐4 and other models to judge creative work, thereby greatly expanding the range of possible tasks available to creativity research.

## AUTHOR CONTRIBUTIONS

**Felix B. Kern:** Conceptualization; investigation; writing – original draft; methodology; visualization; validation; writing – review and editing; software; formal analysis. **Chien‐Te Wu:** Conceptualization; methodology; data curation; investigation; writing – review and editing. **Zenas C. Chao:** Conceptualization; funding acquisition; writing – review and editing; supervision; resources; project administration.

## CONFLICT OF INTEREST STATEMENT

No conflicts of interest.

## Supporting information


**Figure S1.** Comparison of Elo ratings with GPT‐driven ratings using the 3 dimensions, 1 answer approach. (A‐C) Scatterplots directly comparing ratings. (D‐E) Rating distributions (histograms and Gaussian kernel density estimation) in the Novelty and Feasibility dimensions.

**Figure S2.** As above, with the 1 dimension, 10 answers approach.

**Figure S3.** As above, with the 1 dimension, 20 answers approach.

**Figure S4.** As above, with the 1 dimension, 50 answers approach.

**Figure S5.** As above, with the 1 dimension, 20 answers approach with 10 samples.

**Figure S6.** As above, with the 1 dimension, 20 answers, rating‐only approach.

**Figure S7.** As above, with the 1 dimension, 20 answers, rating‐only approach with 10 samples.

**Figure S8.** Distribution of Feasibility ratings across approaches estimated by Gaussian kernel density estimation.

**Figure S9.** Distribution of Spearman’s correlation factors of GPT‐driven versus Elo ratings of Novelty across items. The violin plots estimate the full distribution, the box and whiskers show the quartiles and 10‐90 percentiles, respectively, and the white lines represent the median.

**Figure S10.** Distribution of Spearman’s correlation factors of GPT‐driven versus Elo ratings of Feasibility across items. The violin plots estimate the full distribution, the box and whiskers show the quartiles and 10‐90 percentiles, respectively, and the white lines represent the median.

**Figure S11.** Distribution of Spearman’s correlation factors of GPT‐driven versus Elo ratings of Value across items. The violin plots estimate the full distribution, the box and whiskers show the quartiles and 10‐90 percentiles, respectively, and the white lines represent the median.

**Figure S12.** Goodness of rank‐order fit. Bars display the mean across 30 objects of the root mean squared deviation between the rank order of the Elo ratings and the GPT‐derived ratings of all answers. Error bars represent the 95% confidence interval of the mean.

**Figure S13.** Assessing the impact of the choice fo the scale factor K. Briefly, we recalculated the Elo rating for each item after N matches (i.e., after N binary decisions by a human judge) for all N>1 that had at least 500 answers competing. Since match making was semi‐random for human evaluation, with biases towards answers of similar rating and answers with fewer total matches, not all answers competed equally often; the number of answers competing at least N times is plotted on the right axis in grey. We then calculated the Spearman correlations between the Elo ratings obtained after N matches (curves) or all matches (asterisks) and the ratings obtained by GPT‐4 (1 dimension, 20 answers). The resulting correlations (plotted in colour) are almost indistinguishable across a wide range of K, indicating insensitivity to this parameter.

**Figure S14.** Assessing the stability of the Elo rating approach with respect to match order. We computed the Spearman rank order correlation between the ratings after N matches (see Figure S13) and the ratings obtained by GPT‐4 (1 dimension, 20 answers). These correlations are plotted in black, with an asterisk indicating the final correlation including all matches. We then shuffled the order of matches 20 times, recalculating Elo ratings and plotting the corresponding correlations in colour, again with an asterisk for each resampling indicating the final correlation including all matches. The convergence of the shuffled and original match orders indicates that match order was largely irrelevant to the final outcome, though early scores converged more rapidly in the chosen order due to match making bias.

## Data Availability

The data that support the findings of this study are openly available in OSF at http://doi.org/10.17605/OSF.IO/GDY6C.
