# Supplementary figures and images for: Assessing novelty, feasibility and value of creative ideas with an unsupervised approach using GPT‐4
Source: Br J Psychol. 2024 Jul 22;117(2):741–60. doi: 10.1111/bjop.12720 (PMC13051022; doi:10.1111/bjop.12720)

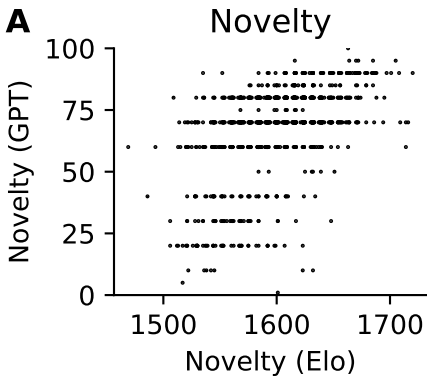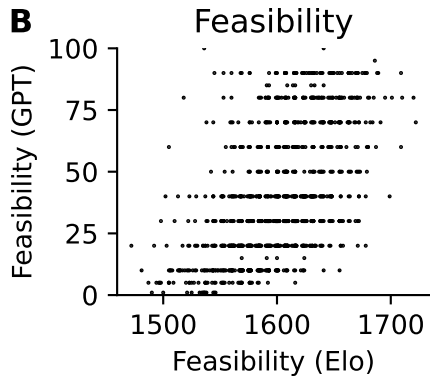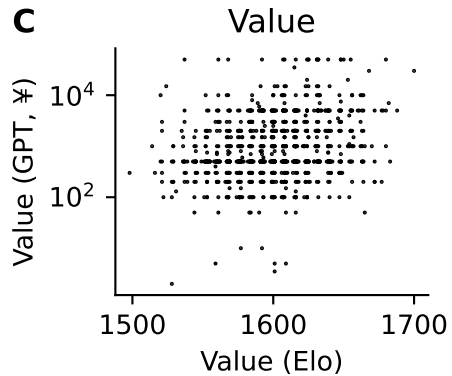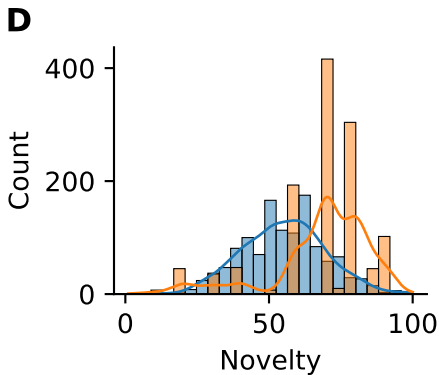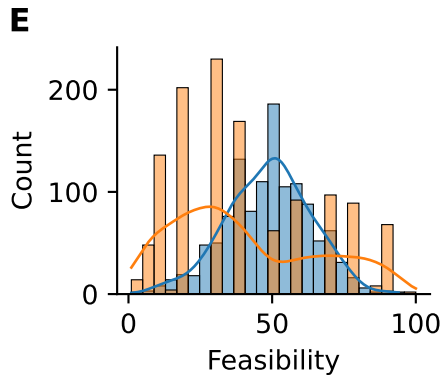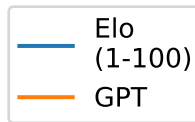

Supplement: Supplementary file 1 — Figure S1. Comparison of Elo ratings with GPT‐driven ratings using the 3 dimensions, 1 answer approach. (A‐C) Scatterplots directly comparing ratings. (D‐E) Rating distributions (histograms and Gaussian kernel density estimation) in the Novelty and Feasibility dimensions. [file BJOP-117-741-s014.pdf]

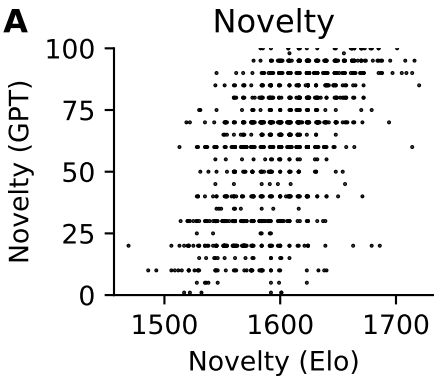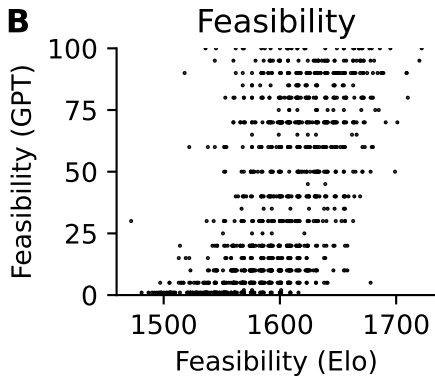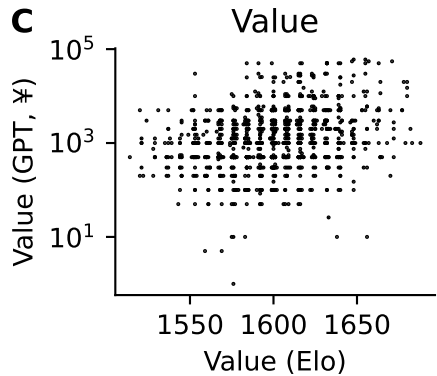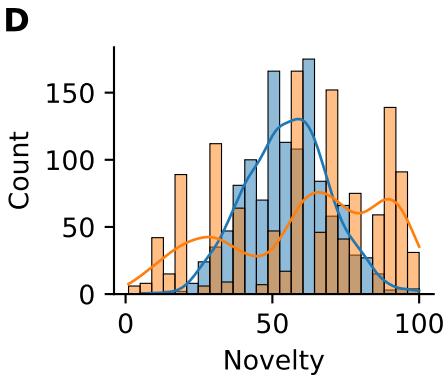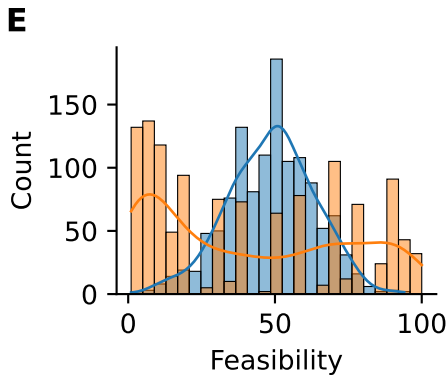

Supplement: Supplementary file 2 — Figure S2. As above, with the 1 dimension, 10 answers approach. [file BJOP-117-741-s001.pdf]

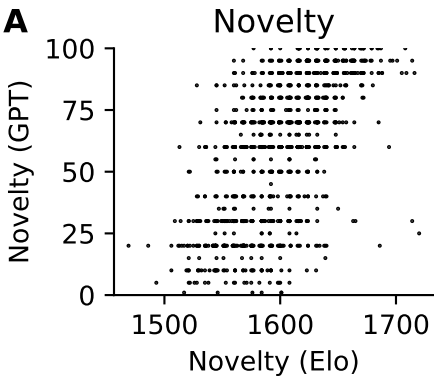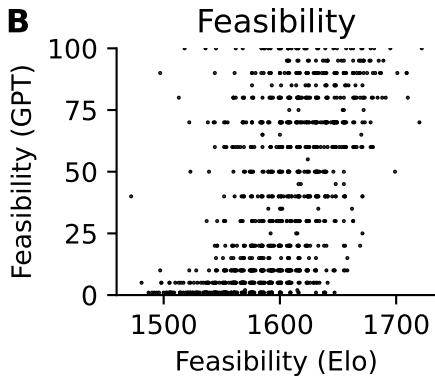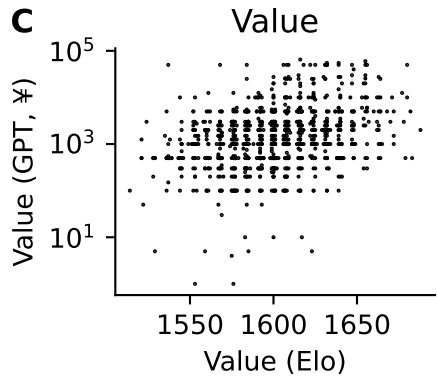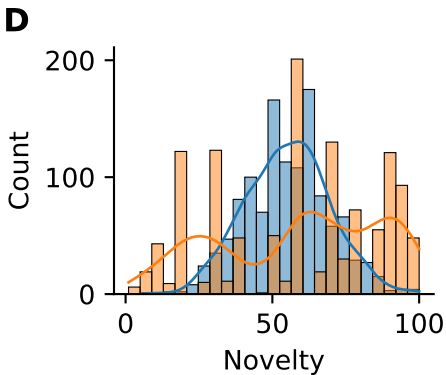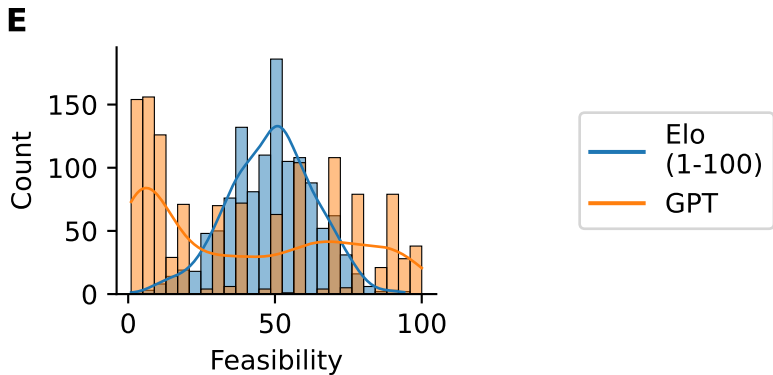

Supplement: Supplementary file 3 — Figure S3. As above, with the 1 dimension, 20 answers approach. [file BJOP-117-741-s013.pdf]

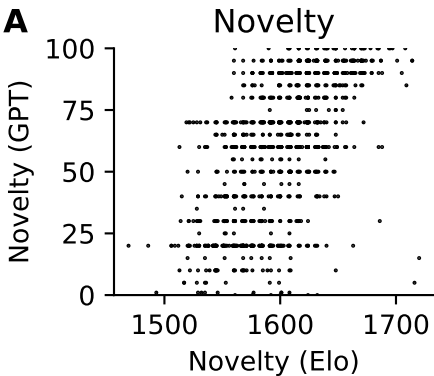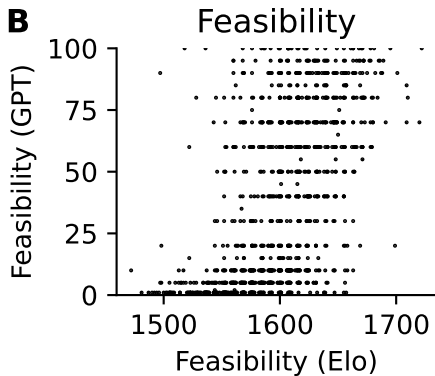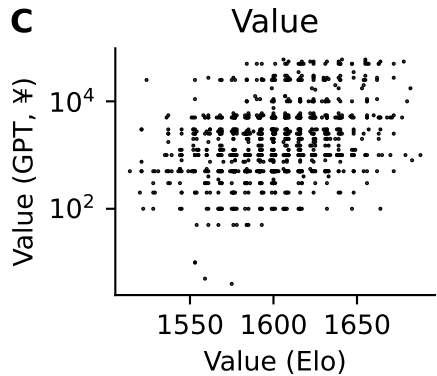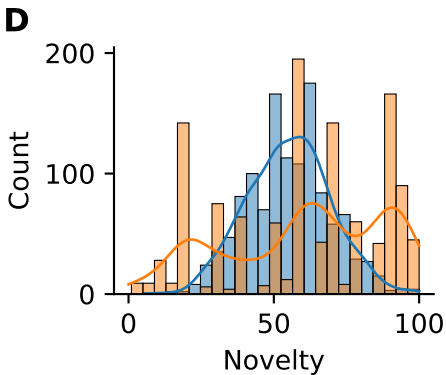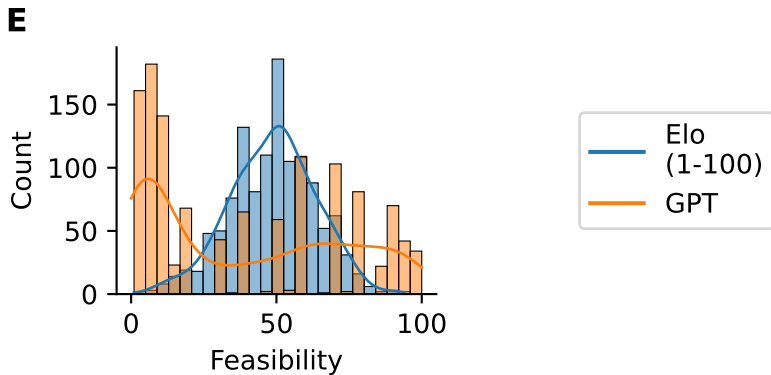

Supplement: Supplementary file 4 — Figure S4. As above, with the 1 dimension, 50 answers approach. [file BJOP-117-741-s008.pdf]

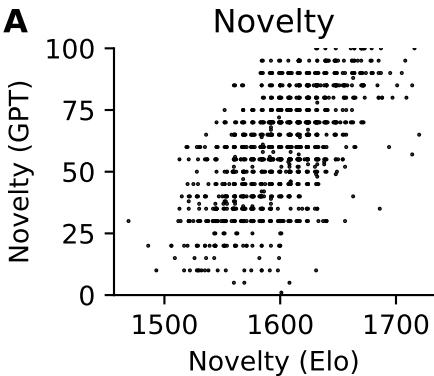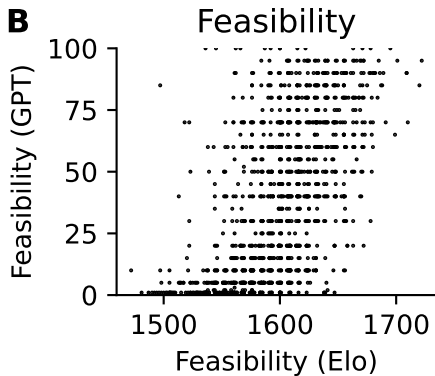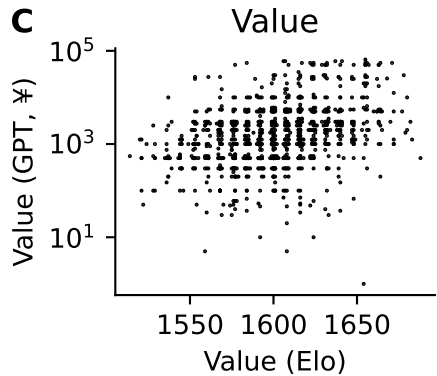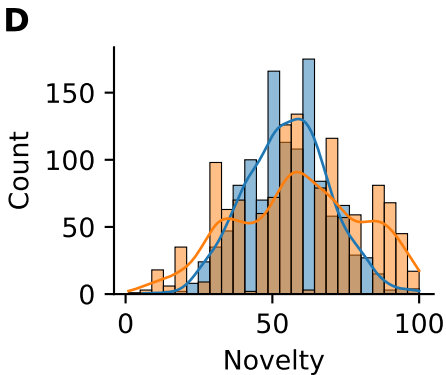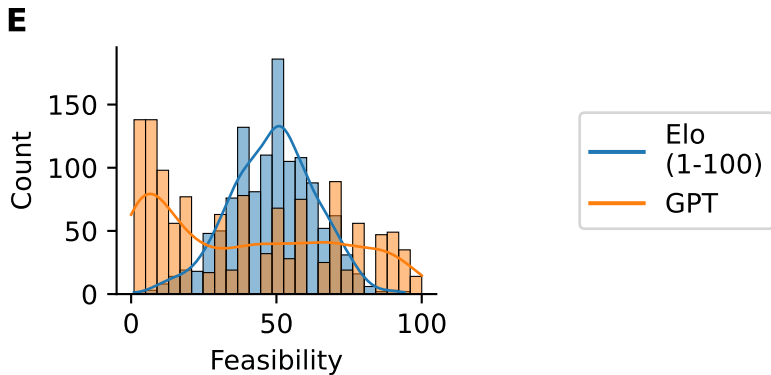

Supplement: Supplementary file 5 — Figure S5. As above, with the 1 dimension, 20 answers approach with 10 samples. [file BJOP-117-741-s002.pdf]

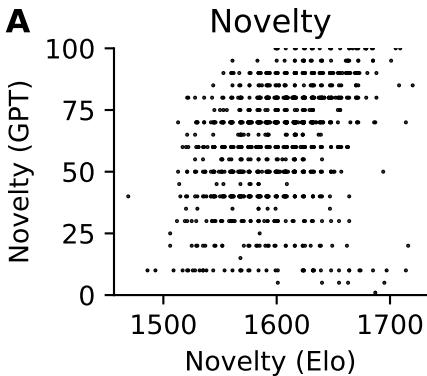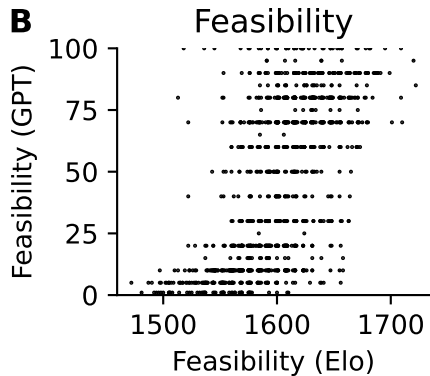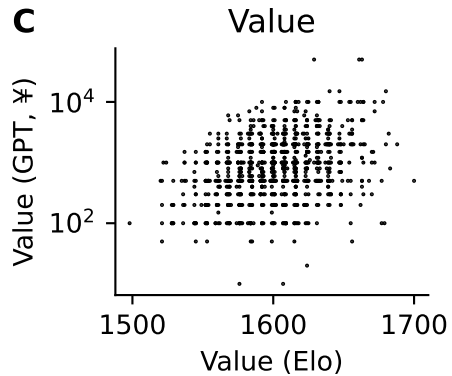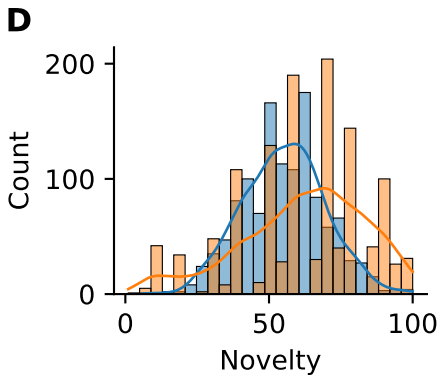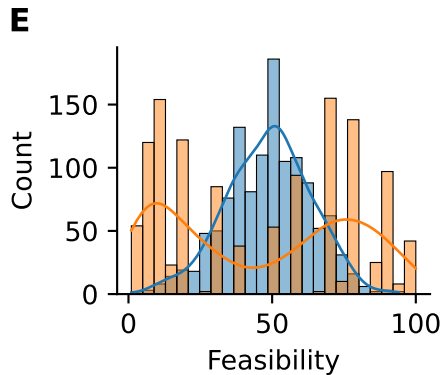

Supplement: Supplementary file 6 — Figure S6. As above, with the 1 dimension, 20 answers, rating‐only approach. [file BJOP-117-741-s003.pdf]

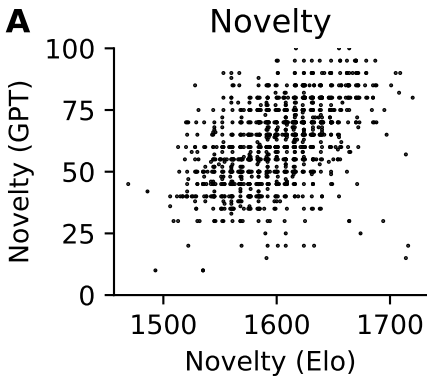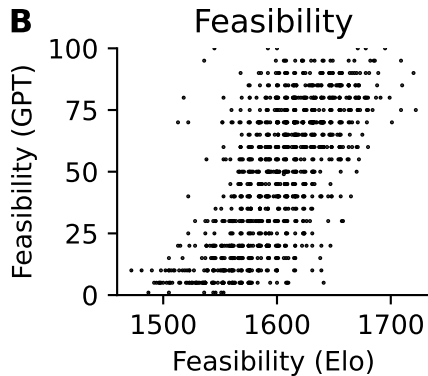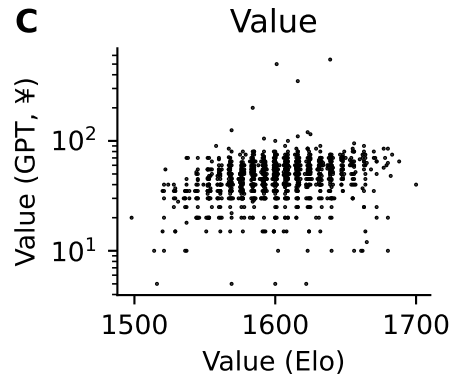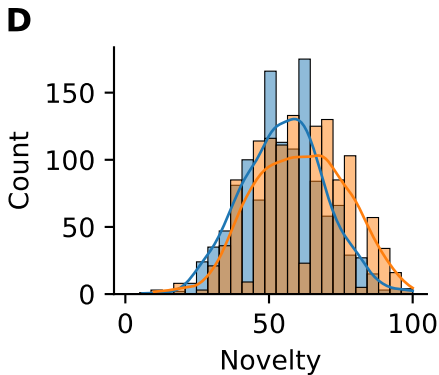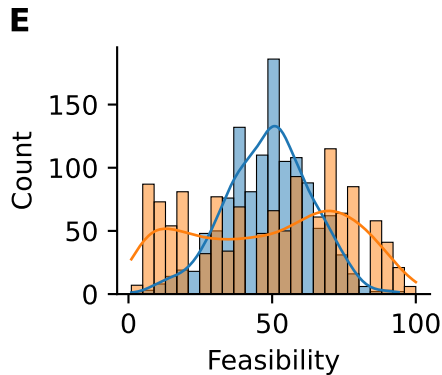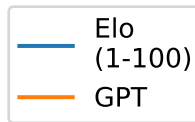

Supplement: Supplementary file 7 — Figure S7. As above, with the 1 dimension, 20 answers, rating‐only approach with 10 samples. [file BJOP-117-741-s005.pdf]

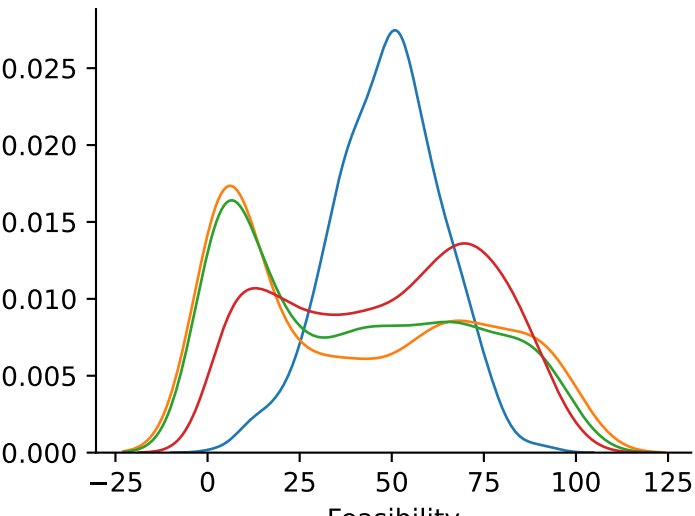

Supplement: Supplementary file 8 — Figure S8. Distribution of Feasibility ratings across approaches estimated by Gaussian kernel density estimation. [file BJOP-117-741-s011.pdf]

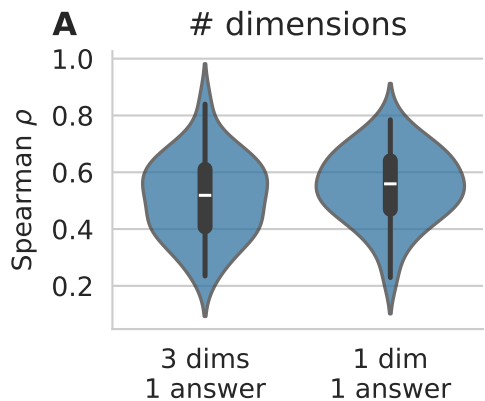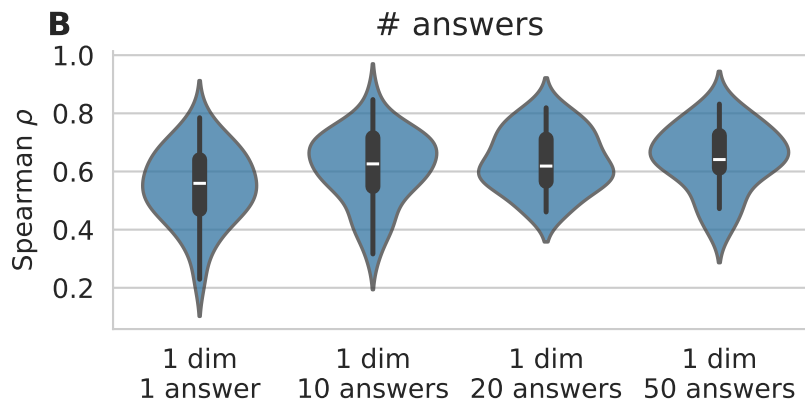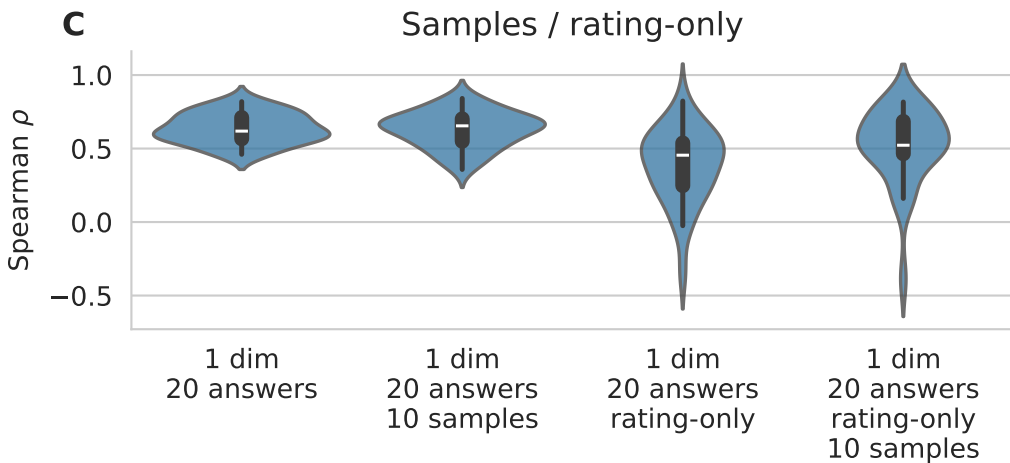

Supplement: Supplementary file 9 — Figure S9. Distribution of Spearman’s correlation factors of GPT‐driven versus Elo ratings of Novelty across items. The violin plots estimate the full distribution, the box and whiskers show the quartiles and 10‐90 percentiles, respectively, and the white lines represent the median. [file BJOP-117-741-s009.pdf]

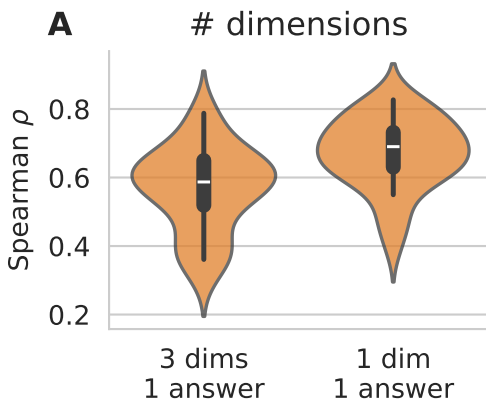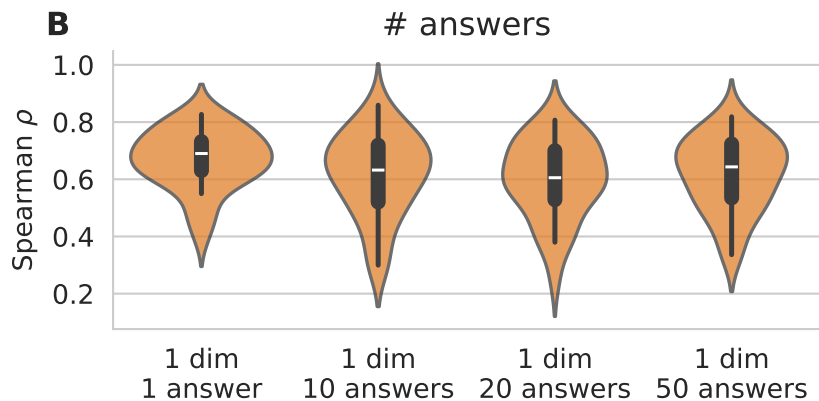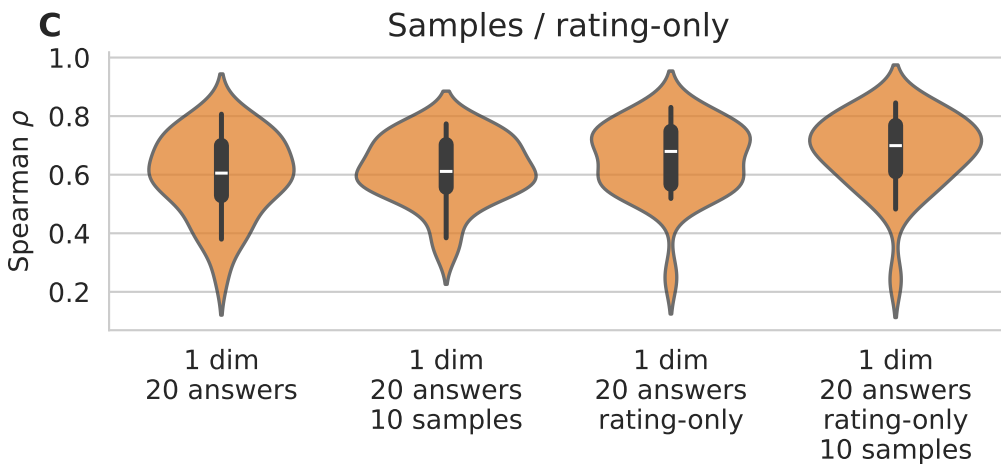

Supplement: Supplementary file 10 — Figure S10. Distribution of Spearman’s correlation factors of GPT‐driven versus Elo ratings of Feasibility across items. The violin plots estimate the full distribution, the box and whiskers show the quartiles and 10‐90 percentiles, respectively, and the white lines represent the median. [file BJOP-117-741-s007.pdf]

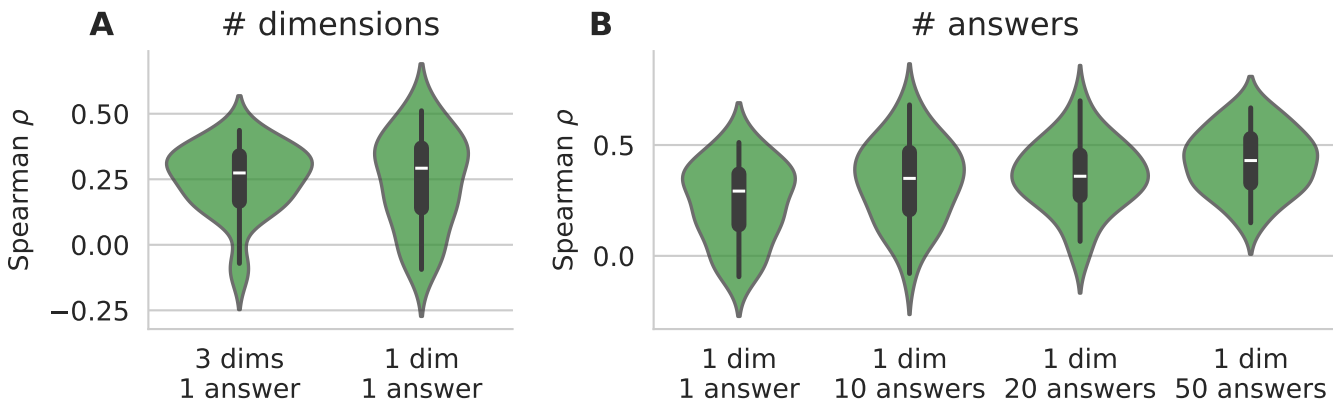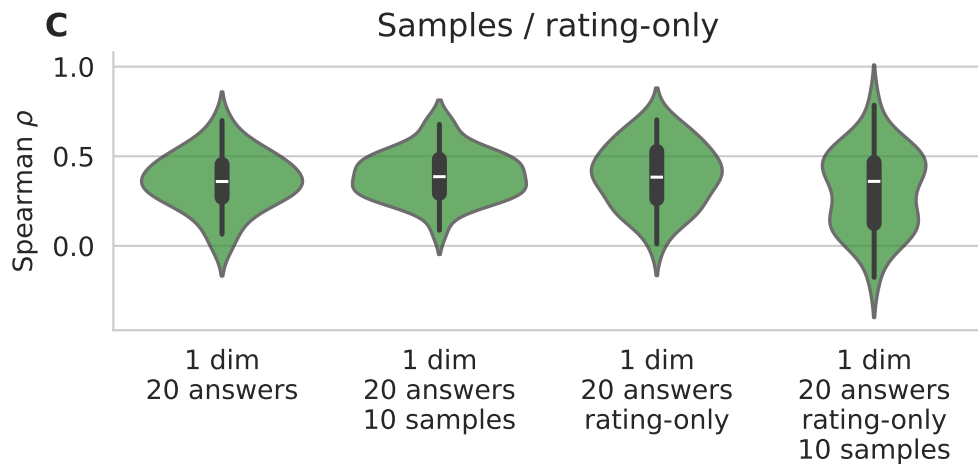

Supplement: Supplementary file 11 — Figure S11. Distribution of Spearman’s correlation factors of GPT‐driven versus Elo ratings of Value across items. The violin plots estimate the full distribution, the box and whiskers show the quartiles and 10‐90 percentiles, respectively, and the white lines represent the median. [file BJOP-117-741-s004.pdf]

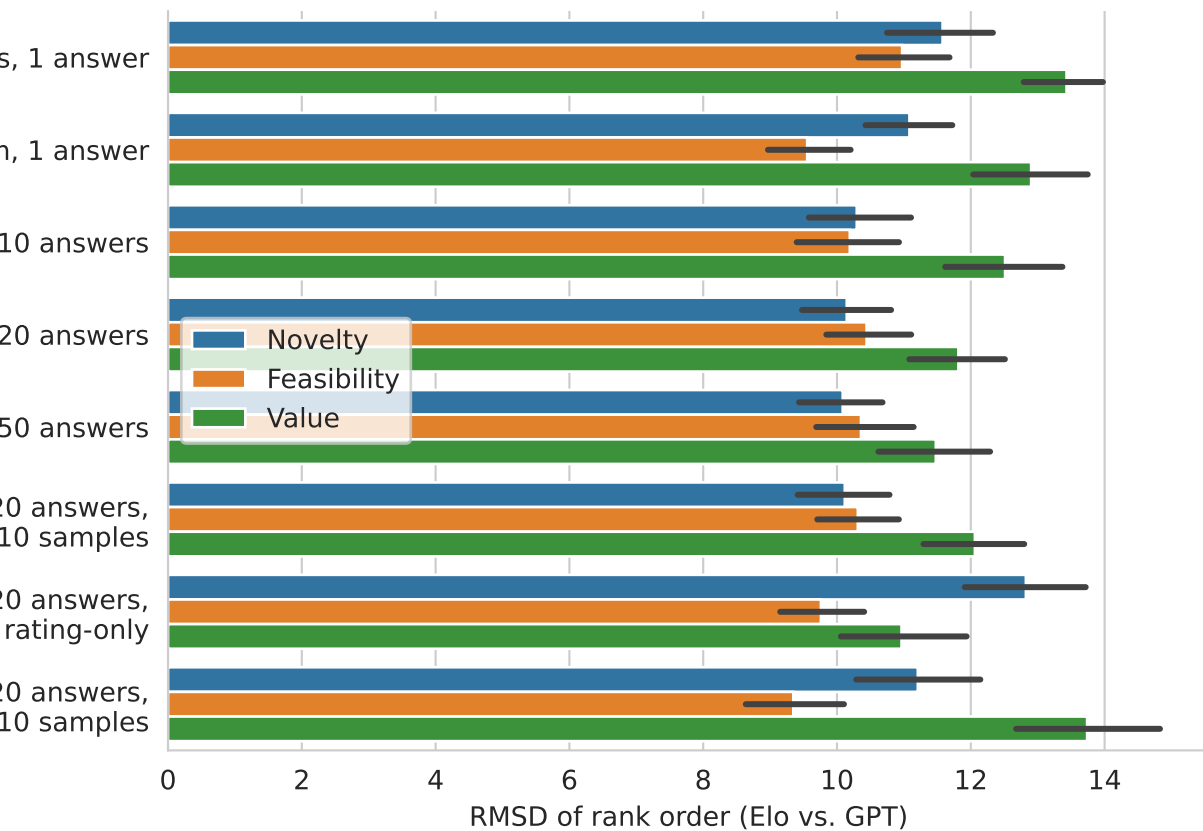

Supplement: Supplementary file 12 — Figure S12. Goodness of rank‐order fit. Bars display the mean across 30 objects of the root mean squared deviation between the rank order of the Elo ratings and the GPT‐derived ratings of all answers. Error bars represent the 95% confidence interval of the mean. [file BJOP-117-741-s012.pdf]

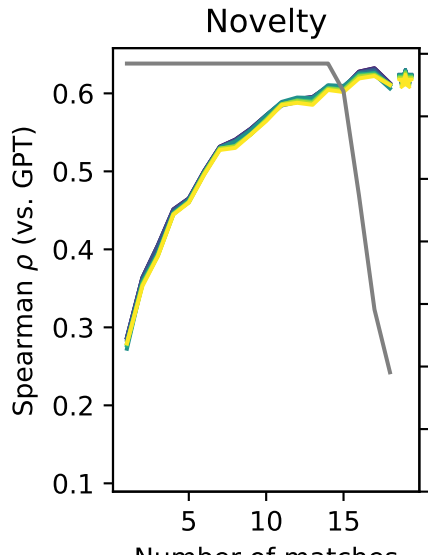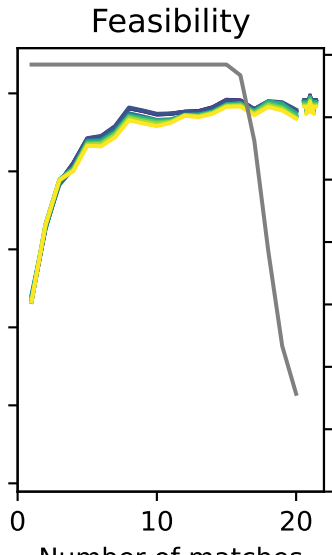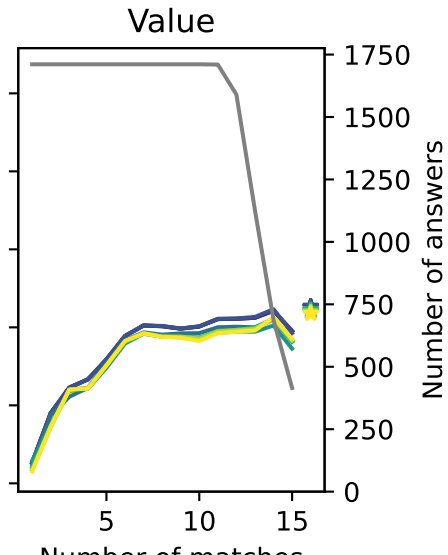

Supplement: Supplementary file 13 — Figure S13. Assessing the impact of the choice fo the scale factor K. Briefly, we recalculated the Elo rating for each item after N matches (i.e., after N binary decisions by a human judge) for all N>1 that had at least 500 answers competing. Since match making was semi‐random for human evaluation, with biases towards answers of similar rating and answers with fewer total matches, not all answers competed equally often; the number of answers competing at least N times is plotted on the right axis in grey. We then calculated the Spearman correlations between the Elo ratings obtained after N matches (curves) or all matches (asterisks) and the ratings obtained by GPT‐4 (1 dimension, 20 answers). The resulting correlations (plotted in colour) are almost indistinguishable across a wide range of K, indicating insensitivity to this parameter. [file BJOP-117-741-s010.pdf]

Novelty

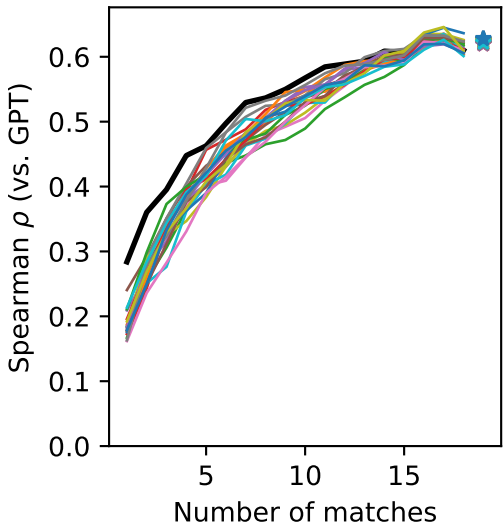

Feasibility

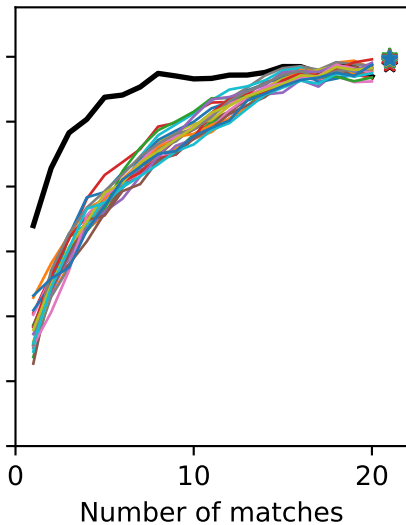

Value

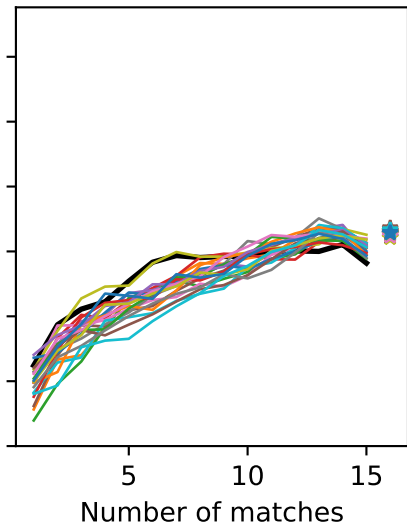

Supplement: Supplementary file 14 — Figure S14. Assessing the stability of the Elo rating approach with respect to match order. We computed the Spearman rank order correlation between the ratings after N matches (see Figure S13) and the ratings obtained by GPT‐4 (1 dimension, 20 answers). These correlations are plotted in black, with an asterisk indicating the final correlation including all matches. We then shuffled the order of matches 20 times, recalculating Elo ratings and plotting the corresponding correlations in colour, again with an asterisk for each resampling indicating the final correlation including all matches. The convergence of the shuffled and original match orders indicates that match order was largely irrelevant to the final outcome, though early scores converged more rapidly in the chosen order due to match making bias. [file BJOP-117-741-s006.pdf]
